# Immune Activation and Inflammation in Patients with Cardiovascular Disease Are Associated with Higher Phenylalanine to Tyrosine Ratios: The Ludwigshafen Risk and Cardiovascular Health Study

**DOI:** 10.1155/2014/783730

**Published:** 2014-02-10

**Authors:** Christian Murr, Tanja B. Grammer, Andreas Meinitzer, Marcus E. Kleber, Winfried März, Dietmar Fuchs

**Affiliations:** ^1^Division of Biological Chemistry, Biocenter, Innsbruck Medical University, Innrain 80, 6020 Innsbruck, Austria; ^2^Medical Clinic V (Nephrology, Hypertensiology, Endocrinolgy, Diabetology, Rheumatology), Mannheim Medical Faculty, University of Heidelberg, 68167 Mannheim, Germany; ^3^Clinical Institute of Medical and Chemical Laboratory Diagnostics, Medical University of Graz, 8010 Graz, Austria; ^4^Synlab Services GmbH, Synlab Academy, 68259 Mannheim, Germany

## Abstract

Higher serum neopterin is associated with increased mortality in patients with coronary artery disease (CAD). Preferentially Th1-type cytokine interferon-**γ** stimulates neopterin production by GTP cychlohydrolase I (GCH-I) in parallel in monocyte-derived macrophages and dendritic cells. In other cells, activation of GCH-I leads to the formation of 5,6,7,8-tetrahydrobiopterin (BH_4_), the necessary cofactor of amino acid hydroxylases like phenylalanine 4-hydroxylase (PAH). Serum concentrations of phenylalanine, tyrosine, neopterin, and high sensitivity C-reactive protein (hsCRP) were measured in 1196 patients derived from the LUdwigshafen RIsk and Cardiovascular Health (LURIC) study, a cohort study among patients referred for coronary angiography. The phenylalanine to tyrosine ratio (Phe/Tyr) served as an estimate of phenylalanine hydroxylase (PAH) enzyme activity. Serum concentrations of phenylalanine and tyrosine and of Phe/Tyr did not differ between individuals with or without CAD (Welch's *t*-test: *P* = n.s.). Higher neopterin and hsCRP concentrations were observed in CAD patients compared to controls (*P* < 0.0001) and they correlated with Phe/Tyr (Spearman's rank correlation for neopterin: *r*_*s*_ = 0.216 and hsCRP: *r*_*s*_ = 0.122; both of *P* < 0.0001) concentrations. In conclusion, immune activation is associated with higher Phe/Tyr in CAD patients. Data indicates subnormal PAH activity which might be involved in the precipitation of neuropsychiatric symptoms in patients.

## 1. Introduction

The development and progression of coronary artery disease (CAD) is closely associated with immune activation and inflammation. Activated macrophages and the proinflammatory cytokine interferon-*γ* (IFN-*γ*) appear to play a major role in these processes [1]. In monocyte-derived macrophages and dendritic cells, IFN-*γ* triggers GTP-cyclohydrolase-I, the key enzyme for the biosynthesis of pteridine derivatives like neopterin and 5,6,7,8-tetrahydrobioptein (BH_4_) [2–4]. Accordingly, the concentration of neopterin serves as a sensitive indicator of Th1-type immune response and is a strong predictor of cardiovascular and total mortality [5–8] which further supports the concept of a prominent role of IFN-*γ* in the pathogenesis of CAD.

There is an indication that inflammation and immune activation also impair the conversion of phenylalanine to tyrosine. Such a relationship has been documented in patients suffering from clinical conditions which go along with immune activation and inflammation such as sepsis, cancer, or HIV-1 infection and also in the healthy elderly in whom increased phenylalanine concentrations and an increased phenylalanine to tyrosine ratio (Phe/Tyr) have been described. Thereby, phenylalanine and Phe/Tyr correlated with markers of immune activation such as neopterin [9–12]. In a pilot study of a small group of patients we recently described similar associations in patients suffering from CAD [13].

In this study, we determined serum concentrations of phenylalanine and tyrosine as well as the Phe/Tyr in 1196 patients within the LURIC cohort in a cross-sectional approach and compared results to concentrations of neopterin and to the inflammation marker high sensitivity C-reactive protein (hsCRP). To account for kidney function and to calculate glomerular filtration rate (GFR), concentrations of creatinine and cystatin C were included in this analysis.

## 2. Patients and Methods

### 2.1. Subjects

Samples of 3316 Caucasian patients hospitalized for angiography were collected from June 1997 to January 2000 [14]. Informed written consent was obtained from each of the participants, and the study was approved by the ethics review committee at the Landesärztekammer Rheinland-Pfalz. Clinically relevant coronary artery disease (CAD) was defined as the occurrence of a visible luminal narrowing that is equal to or greater than 20 percent stenosis in at least 1 of 15 coronary segments according to the classification of the American Heart Association [15]. Angiograms were analyzed as described previously [14].

All patients with complete data sets available for phenylalanine and tyrosine, neopterin, hsCRP, creatinine, and cystatin C were included in the study resulting in 1196 patients (824 males, 372 females; = 36% of the total LURIC cohort) with a median age of 63.4 years (interquartile range: 56.0–70.6, range: 18.5–92.1 years). There was no statistically significant difference to the excluded group concerning age (mean ± SD: 62.7 ± 10.6 years; Welch's *t*-test: *P* = n.s.) or distribution of CAD (Fisher's exact test: *P* = n.s.).

### 2.2. Measurements

Blood samples were drawn by venous puncture in the morning before cardiac catheterization, after subjects had fasted. The blood was allowed to clot at room temperature, and serum was obtained by centrifugation at 3220 ×g for 15 minutes. One mL aliquots of serum was shock frozen in liquid nitrogen and stored at −80°C for later use.

Concentrations of phenylalanine and tyrosine in serum were measured with a conventional amino acid analysis technique, involving separation of amino acids by ion exchange chromatography followed by postcolumn continuous reaction with ninhydrin [16, 17]. Within-day and between-day coefficients of variation (CV) at different concentrations were below 9% throughout. Phe/Tyr was calculated to estimate the activity of the enzyme phenylalanine hydroxylase (PAH) [9, 18, 19]. Creatinine (conversion to SI units: mg/dL × 88.4 = *μ*mol/L) was measured by the method of Jaffé at 37°C; total cholesterol (conversion to SI units: mg/dL × 0.02586 = mmol/L) and triglycerides (conversion to SI units: mg/dL × 0.0114 = mmol/L) by enzymatic assays from DiaSys (Holzheim, Germany) on an Olympus AU640 automatic analyzer LDL- and HDL-cholesterol were determined in EDTA plasma by a combined ultracentrifugation and precipitation method. The standard laboratory methods and blood pressure measurements have been described earlier [14].

Cystatin C and hsCRP were measured by immunonephelometry (N High Sensitive CRP; N Latex Cystatin C, Siemens, formerly Dade Behring, Marburg, Germany). Serum neopterin concentrations were determined by a commercially available radioimmunoassay (BRAHMS Diagnostica, Hennigsdorf, Germany) with a sensitivity of 1 nmol/L neopterin and an interassay coefficient of variation ranging from 3.9% to 8.2% [20]. GFR was calculated on the basis of serum creatinine, cystatin C, age, and gender by the following equation: GFR (mL/min/1.73 m²) = 177.6 × [Creatinine (mg/dL)]^−0.65^  ×  [Cystatin C (mg/L)]^−0.57^  ×  Age^−0.20^  ×  (0.82 if female) (described in [21]).

### 2.3. Statistical Analysis

Patients were stratified according to the presence or absence of CAD. Frequencies between subgroups were compared by Fisher's exact test. Differences of mean laboratory variables among patient groups with and without CAD were tested for significance by Welch's *t*-test, which does not assume equal variances. Correlation between variables was assessed by the nonparametric Spearman's rank correlation test, which was done with the program GraphPad Prism (GraphPad Software, Inc., San Diego, CA). For these calculations, the following assumptions concerning CAD were made: no CAD (0–10% stenosis) = 0, 11–49% stenosis = 1, one vessel disease = 2, and two- or three-vessel disease = 3. Acute coronary syndrome (ACS) was classified as: no ACS = 0, unstable angina pectoris = 1, non-ST-elevation myocardial infarction (NSTEMI) = 2, and ST-elevation myocardial infarction (STEMI) = 3.

We examined the relationship between Phe/Tyr, neopterin, hsCRP, calculated GFRs, systolic and diastolic blood pressure, ACS scores, sex, body mass index (BMI) and age by linear regression analysis using a stepwise version of a linear regression model as implemented in the program SPSS 11.0 for Windows (SPSS, Inc., Chicago, IL). This technique identifies the subset of variables that predict the dependent variable in the best way. We used forward variable selection with “*P*-to-enter” set at 0.05, and the “*P*-to-remove” at 0.10.

## 3. Results

Among the 1196 study subjects, 20.8% had no CAD (control group), whereas 79.2% presented with CAD; 87% of the studied cohort reported on antihypertensive drug medication. As seen from Table 1, there was no statistically significant difference of phenylalanine, tyrosine, Phe/Tyr, triglycerides, mean diastolic blood pressure and body mass index between the controls and those with CAD (Welch's *t* test: *P* = n.s.). Patients with CAD had higher neopterin and hsCRP concentrations and higher systolic blood pressures but lower calculated GFRs and total, HDL- and LDL cholesterol concentrations than the control group.

**Figure 1:**
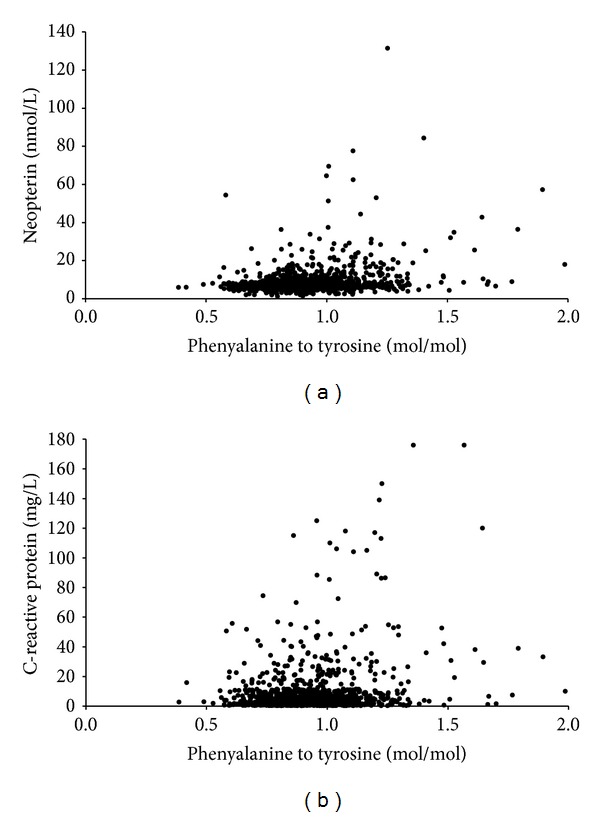
Scatter plots of phenylalanine to tyrosine ratio versus neopterin (a) or hsCRP (b) concentrations of the investigated 1196 subjects; Spearman's rank correlation coefficients *r*_*s*_ for neopterin = 0.216 and hsCRP = 0.122; both of  *P* < 0.0001.

**Figure 2:**
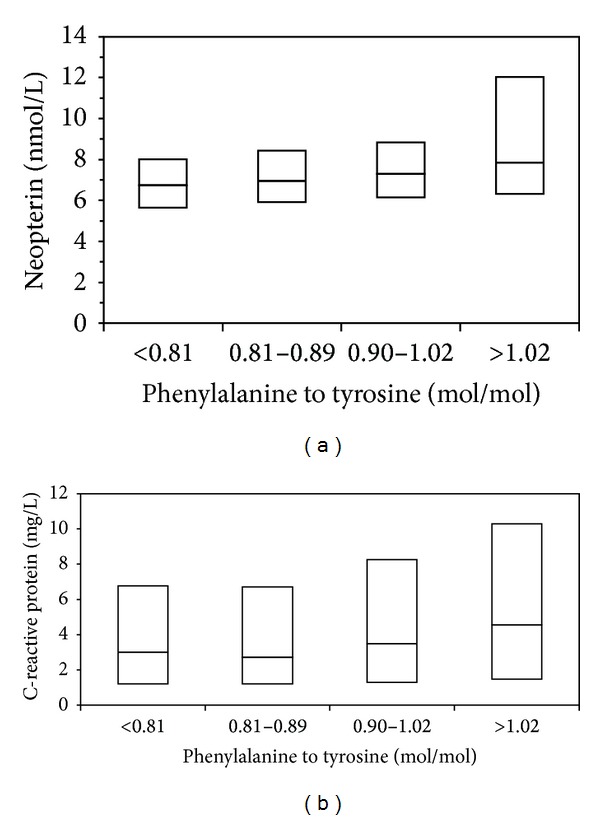
Box plots of serum neopterin (a) and hsCRP (b) concentrations of patients according to quartiles of phenylalanine to tyrosine ratios. The boxes extend from the 25th percentile to the 75th percentile, with a horizontal line at the median (50th percentile); all two observations: *P* < 0.001 (Kruskal-Wallis test).

Phe/Tyr increased with age and was positively correlated with serum neopterin, hsCRP, and ACS score, but inversely correlated with calculated GFRs, body mass index, diastolic and systolic blood pressure, and the number of daily smoked cigarettes (Table 2). There was no significant correlation of Phe/Tyr with total cholesterol, LDL- and HDL-cholesterol, and triglycerides concentrations or with the extent of CAD. Among the variables which revealed a significant association with Phe/Tyr, the calculated GFRs (*r*_*s*_ = −0.264), neopterin (*r*_*s*_ = 0.216), hsCRP (*r*_*s*_ = 0.122), and BMI (*r*_*s*_ = −0.135) had the strongest relationship.  Figure 1 depicts the association of Phe/Tyr with neopterin and hsCRP concentrations. In Figure 2 neopterin concentrations and hsCRP concentrations are shown according to the quartiles of Phe/Tyr.

To examine the relationship between Phe/Tyr, neopterin, hsCRP, calculated GFRs, systolic and diastolic blood pressure, ACS score, sex, BMI, and age, linear regression analysis was done. When a stepwise regression was performed, including all univariately significant variables as candidates, all variables with the exception of ACS score, sex, and diastolic and systolic blood pressure were included in the model. Thereby, the following equation resulted: Phe/Tyr = 1.338 + 0.0029 × [Neopterin] + 0.002 × [CRP] − 0.0022 × GFR − 0.0016 × Age − 0.0057 × BMI − 0.0008 × Cigarettes per day. By this equation about 16% of the variation of the Phe/Tyr could be explained (*n* = 1196, *r* = 0.405, *r* ^2^ = 0.164, *P* < 0.001). As calculated from a *t*-statistics, all the variables, neopterin (*t* = 3.94, *P* < 0.001), and hsCRP concentrations (*t* = 7.20, *P* = *P* < 0.001), GFR (*t* = −7.59, *P* < 0.001), BMI (*t* = −4.64, *P* < 0.001), age (*t* = −3.15, *P* = 0.002), and the number of daily smoked cigarettes (*t* = −2.44, *P* = 0.015), significantly contributed to Phe/Tyr which indicates an independent influence of these variables.

## 4. Discussion

In a large cohort of patients referred for coronary angiography we have observed that serum Phe/Tyr concentrations are associated with the inflammatory markers neopterin and CRP but are negatively correlated with glomerular filtration rate, whereas there was no significant association with the presence of CAD. The positive correlation found between Phe/Tyr and neopterin agrees with the idea that low PAH functional activity is related to Th1-type immune activation. Similar positive relationships between Phe/Tyr and immune activation markers have been described earlier in patients suffering from diseases in which immune activation plays a major role and is associated with adverse outcomes like ovarian carcinoma, HIV-1 infection, and also in patients after trauma and with sepsis [10, 11, 22]. Moreover, recently in patients with hepatitis C-virus infection under treatment with IFN-*α*/ribavirin, an increase of phenylalanine and Phe/Tyr concentrations was reported [23, 24]. Taking all the results together it seems quite reasonable that immune activation leads to the increase of Phe/Tyr in patients. The inverse correlation of Phe/Tyr with glomerular filtration rate may be explained by the fact that the enzyme PAH is not only located in liver cells but also in the kidney [25] and impaired renal function may also alter PAH enzyme activity.

The diminished conversion of phenylalanine to tyrosine by PAH may be due to an increased output of reactive oxygen species (ROS) which is produced by macrophages upon stimulation by IFN-*γ* in parallel to neopterin production [26]. Consequently, neopterin concentration can also serve as a surrogate indicator of oxidative stress due to immune activation [27, 28]. Recently, in a molecular dynamics study of PAH a potential molecular mechanism of enzyme downregulation upon oxidative stress by modulation of the local dynamics of a loop region near the active site was demonstrated [29].

In general, oxidative stress may interfere with the tertiary structure of proteins by oxidation of sulfhydryl groups. Such structural changes may downregulate catalytic activity as was demonstrated recently for the reduction of disulphide-bridges in human ß-defensin 1 (hBD-1) which becomes a potent antimicrobial peptide against the opportunistic pathogenic fungus *Candida albicans* and against anaerobic, Gram-positive commensals of *Bifidobacterium *and *Lactobacillus* species [30]. Thereby, it was evident that reduced hBD-1 differs structurally from oxidized hBD-1 and free cysteines in the carboxy terminus seem to be important for the bactericidal effect.

The increase of Phe/Tyr in CAD patients may be due to impaired function of PAH resulting from BH_4_ deficiency. As a consequence of the very limited possibilities to determine BH_4_ concentrations directly in clinical settings, the monitoring of Phe/Tyr was proposed as an indirect marker of BH_4_ functional activity [13]. During inflammatory processes involving increased formation of IFN-*γ*, GTP-cyclohydrolase I is induced and primarily leads to the formation of BH_4_. In humans and primates only a significant production of neopterin is detected in cells of the monocytes-macrophages lineage, whereas in other cells and in other species only the biosynthesis of BH_4_ is of relevance [20]. BH_4_, the necessary cofactor of several monooxygenases of amino acids including PAH [31], nitric oxide (NO) synthases (NOS) [32], and of glycerylether monoxygenase [33], is released in increased amounts from, for example, endothelial cells. Loss or oxidation of BH_4_ to 7,8-dihydrobiopterin (BH_2_) is associated with NOS uncoupling that results in the production of vasoconstrictory superoxide anion (O_2_^−^, hyperoxide anion) at the expense of vasodilatory NO [34]. As a consequence, production of vasodilatory NO falls during chronic immune activation processes and blood pressure rises. Interestingly in our study no association of neopterin and blood pressure values was found (not shown), and a significant albeit unexpected association of higher Phe/Tyr with lower blood pressure was observed. Thereby the high frequency of antihypertensive drug medication in our study could play a role.

According to the literature, alterations of phenylalanine and of Phe/Tyr could be involved in a wide spectrum of neuropsychiatric abnormalities [9, 12, 35]. In healthy elderly individuals, increased phenylalanine concentrations and increased Phe/Tyr have been found to be correlated with scores for depression like the Montgomery-Asberg Depression Rating Scale (MADRS) or with a fatigue inventory or with the Neurotoxicity Rating Scale (NRS) [12]. Additionally, the increase of blood Phe/Tyr and phenylalanine concentrations in patients with HCV infection under treatment with IFN-*α* was reported to be associated with low dopamine concentrations in the cerebrospinal fluid and correlated with fatigue scores [24]. Unfortunately, neuropsychiatric test results were not available from our patients, and further studies are to be conducted to address this relationship in patients with CAD.

In conclusion, our study provides data that patients with CAD and higher serum neopterin or CRP levels may exhibit higher Phe/Tyr, an abnormality which is most probably due to impaired PAH activity. However, results need to be confirmed by further investigation. Still, our data indicate disturbed phenylalanine metabolism in patients which could possibly be associated with neuropsychiatric disturbances, because phenylalanine metabolism is closely related to neurotransmitter biosynthesis.

## Figures and Tables

**Table 1:** Characteristics of coronary artery disease (CAD) patients and controls (*n* = 1196) given as mean ± SD; n.s.: not significant (Welch's *t* test), GFR: glomerular filtration rate, hsCRP: high sensitivity C-reactive protein.

Variable	Controls	CAD	*T* value	*P* value
	(*n* = 249)	(*n* = 947)		
Age, years	58.4 ± 12.3	63.6 ± 10.0	−6.26	<0.0001
Neopterin, nmol/L	7.6 ± 4.7	9.1 ± 8.2	−3.76	<0.0001
hsCRP, mg/L	5.8 ± 7.6	9.3 ± 18.5	−3.88	<0.0001
Creatinine, mg/dL	0.88 ± 0.32	0.99 ± 0.46	−4.11	<0.0001
Cystatin C, mg/L	0.9 ± 0.3	1.0 ± 0.4	−4.04	<0.0001
GFR, mL/min/1.73 m^2^	87.6 ± 19.5	81.6 ± 20.9	4.21	<0.0001
Phenyalanine, *µ*mol/L	57.4 ± 12.2	58.2 ± 12.1	−0.97	n.s.
Tyrosine, *µ*mol/L	64.9 ± 16.3	64.4 ± 16.0	0.47	n.s.
Phenylalanine/tyrosine, mol/mol	0.91 ± 0.17	0.93 ± 0.18	−1.91	n.s.
Total cholesterol, mg/dL	217 ± 42	206 ± 44	3.90	<0.0001
LDL-cholesterol, mg/dL	121 ± 32	116 ± 35	2.50	0.0128
HDL-cholesterol, mg/dL	43 ± 11	37 ± 10	7.27	<0.0001
Triglycerides, mg/dL	165 ± 178	171 ± 102	−0.51	n.s.
Systolic blood pressure, mm Hg	136 ± 21	142 ± 24	−3.79	<0.0001
Diastolic blood pressure, mm Hg	80 ± 11	81 ± 12	−1.33	n.s.
Daily smoked cigarettes, *n*	9.0 ± 15	14 ± 16	−4.87	<0.0001
Body mass index, kg/m^2^	26.8 ± 4.0	27.2 ± 3.9	−1.41	n.s.

GFR (mL/min/1.73 m^2^) = 177.6 ×  [Creatinine]^−0.65^ × [Cystatin C]^−0.57^  × Age^−0.20^  × (0.82 if female); conversion factors to SI units: creatinine: mg/dL × 88.4 = *µ*mol/L; cholesterol: mg/dL × 0.02586 = mmol/L; triglycerides: mg/dL × 0.0114 = mmol/L; and blood pressure: mm Hg × 0.133 = kPa.

**Table 2:** Spearman's rank correlations of investigated characteristics (*n* = 1196).

Phenylalanine to tyrosine ratio versus	Spearman's rank correlations *r*_*s*_ value (95% confidence interval)	*P* value
Neopterin	0.216 (0.159–0.271)	<0.0001
hsCRP	0.122 (0.064–0.179)	<0.0001
ACS score	0.100 (0.042–0.157)	0.0005
Age	0.098 (0.040–0.156)	0.0007
CAD score	0.048 (−0.011–0.106)	n.s.
HDL-cholesterol	−0.010 (−0.068–0.049)	n.s.
Metabolic syndrome score	−0.016 (−0.074–0.043)	n.s.
Triglycerides	−0.034 (−0.092–0.025)	n.s.
LDL-cholesterol	−0.040 (−0.098–0.019)	n.s.
Total cholesterol	−0.045 (−0.103–0.014)	n.s.
Systolic blood pressure	−0.058 (−0.116–0.000)	0.0434
Daily smoked cigarettes	−0.059 (−0.117–−0.001)	0.0412
Diastolic blood pressure	−0.110 (−0.167–−0.051)	0.0001
BMI	−0.135 (−0.192–−0.077)	<0.0001
GFR	−0.264 (−0.318–−0.209)	<0.0001

GFR: glomerular filtration rate, hsCRP: high sensitivity C-reactive protein, and n.s.: not significant; coronary artery disease (CAD) score: 0: no CAD; 1: 11 to 49% stenosis; 2: one vessel disease; 3: two- or three-vessel disease; acute coronary syndrome (ACS) score: 0: no ACS; 1: unstable angina pectoris; 2: NSTEMI; 3: STEMI.
